# Supplementary material for: Nutritional Composition of Honey Bee Drones of Two Subspecies Relative to Their Pupal Developmental Stages
Source: Insects. 2021 Aug 23;12(8):759. doi: 10.3390/insects12080759 (PMC8396957; doi:10.3390/insects12080759)
Supplement: Supplementary file 1 [file insects-12-00759-s001.zip › insects-1335852-supplementary.pdf]

Table S1. Alignment report of *Apis mellifera carnica* (AMC) and *Apis mellifera mellifera* (AMM) COI gene.

```
Majority -----GTTCATCAATGAGACTTATTATTCGAATAGAATTAAGATCTCCAGGATCATGAATTAATAACGATCAAATTTATA
---------+---------+---------+---------+---------+---------+---------+---------+
         10        20        30        40        50        60        70        80
---------+---------+---------+---------+---------+---------+---------+---------+
AMC_1 ---------ATCAATGAGACTTATTATTCGAATAGAATTAAGATCCCCAGGATCATGAATTAGCAATGATCAAATTTATA 71
AMC_2 ACTAGGATCATCAATGAGACTTATTATTCGAATAGAATTAAGATCCCCAGGATCATGAATTAACAATGATCAAATTTATA 80
AMM_1 ---AGGTTCATCAATGAGACTTATTATTCGAATAGAATTAAGATCTCCAGGATCATGAATTAATAACGATCAAATTTATA 77
AMM_2 -----GTTCATCAATGAGACTTATTATTCGAATAGAATTAAGATCTCCAGGATCATGAATTAATAACGATCAAATTTATA 75
AMM_3 -------TCATCAATGAGACTTATTATTCGAATAGAATTAAGATCTCCAGGATCATGAATTAATAACGATCAAATTTATA 73

Majority ATACAATTGTTACTAGCCACGCATTTCTAATAATCTTTTTTATAGTTATACCATTTCTAATTGGAGGATTTGGAAATTGG
         ---------+---------+---------+---------+---------+---------+---------+---------+
                 90       100       110       120       130       140       150       160
         ---------+---------+---------+---------+---------+---------+---------+---------+
AMC_1    ATACAATTGTTACTAGTCATGCATTCCTAATAATTTTTTTTATAGTTATACCATTTTTAATTGGAGGATTTGGAAATTGG 151
AMC_2    ATACAATTGTTACTAGTCATGCATTCCTAATAATTTTTTTTATAGTTATACCATTTTTAATTGGAGGATTTGGAAATTGG 160
AMM_1    ATACAATTGTTACTAGCCACGCATTTCTAATAATCTTTTTTATAGTTATACCATTTCTAATTGGAGGATTTGGAAATTGG 157
AMM_2    ATACAATTGTTACTAGCCACGCATTTCTAATAATCTTTTTTATAGTTATACCATTTCTAATTGGAGGATTTGGAAATTGG 155
AMM_3    ATACAATTGTTACTAGCCACGCATTTCTAATAATCTTTTTTATAGTTATACCATTTCTAATTGGAGGATTTGGAAATTGG 153

Majority CTTATTCCTTTAATACTAGGATCACCTGATATAGCATTTCCCCGAATAAATAATGTTAGATTTTGATTACTTCCTCCCTC
         ---------+---------+---------+---------+---------+---------+---------+---------+
                170       180       190       200       210       220       230       240
         ---------+---------+---------+---------+---------+---------+---------+---------+
AMC_1    CTTATTCCTTTAATACTAGGATCACCTGATATAGCATTCCCCCGAATAAATAATATTAGATTTTGATTACTTCCTCCCTC 231
AMC_2    CTTATTCCTTTAATACTAGGATCACCTGATATAGCATTCCCCCGAATAAATAATATTAGATTTTGATTACTTCCTCCCTC 240
AMM_1    CTTATTCCTTTAATACTAGGATCACCTGATATAGCATTTCCCCGAATAAATAATGTTAGATTTTGATTACTTCCTCCCTC 237
AMM_2    CTTATTCCTTTAATACTAGGATCACCTGATATAGCATTTCCCCGAATAAATAATGTTAGATTTTGATTACTTCCTCCCTC 235
AMM_3    CTTATTCCTTTAATACTAGGATCACCTGATATAGCATTTCCCCGAATAAATAATGTTAGATTTTGATTACTTCCTCCCTC 233

Majority ATTATTAATACTTTTATTAAGAAATTTATTTTACCCAAGACCAGGAACTGGATGAACAGTATATCCACCATTATCAGCAT
```

---------+---------+---------+---------+---------+---------+---------+---------+

250 260 270 280 290 300 310 320

---------+---------+---------+---------+---------+---------+---------+---------+

AMC_1 ATTATTTATACTTTTATTAAGAAATTTATTTTATCCAAGACCAGGAACTGGATGAACAGTATATCCACCATTATCAGCAT 311
AMC_2 ATTATTTATACTTTTATTAAGAAATTTATTTTATCCAAGACCAGGAACTGGATGAACAGTATATCCACCATTATCAGCAT 320
AMM_1 ATTATTAATACTTTTATTAAGAAATTTATTTTACCCAAGACCAGGAACTGGATGAACAGTATATCCACCATTATCAGCAT 317
AMM_2 ATTATTAATACTTTTATTAAGAAATTTATTTTACCCAAGACCAGGAACTGGATGAACAGTATATCCACCATTATCAGCAT 315
AMM_3 ATTATTAATACTTTTATTAAGAAATTTATTTTACCCAAGACCAGGAACTGGATGAACAGTATATCCACCATTATCAGCAT 313

Majority ATTTATATCATTCTTCACCTTCAGTAGATTTTGCAATTTTTTCTCTTCATATATCAGGAATTTCCTCAATTATAGGATCA

---------+---------+---------+---------+---------+---------+---------+---------+

330 340 350 360 370 380 390 400

---------+---------+---------+---------+---------+---------+---------+---------+

AMC_1 ATTTATATCATTCTTCACCTTCAGTAGATTTTGCAATTTTTTCTCTTCATATATCAGGAATTTCCTCAATTATAGGATCA 391
AMC_2 ATTTATATCATTCTTCACCTTCAGTAGATTTTGCAATTTTTTCTCTTCATATATCAGGAATTTCCTCAATTATAGGATCA 400
AMM_1 ATTTATATCATTCTTCACCTTCAGTAGATTTTGCAATTTTTTCTCTTCATATATCAGGAATTTCCTCAATTATAGGATCA 397
AMM_2 ATTTATATCATTCTTCACCTTCAGTAGATTTTGCAATTTTTTCTCTTCATATATCAGGAATTTCCTCAATTATAGGATCA 395
AMM_3 ATTTATATCATTCTTCACCTTCAGTAGATTTTGCAATTTTTTCTCTTCATATATCAGGAATTTCCTCAATTATAGGATCA 393

Majority TTAAATTTAATAGTTACAATTATAATAATAAAAAATTTTTCTATAAATTATGACCAAATTTCATTATTTCCATGATCAGT

---------+---------+---------+---------+---------+---------+---------+---------+

410 420 430 440 450 460 470 480

---------+---------+---------+---------+---------+---------+---------+---------+

AMC_1 TTAAACTTAATAGTTACAATTATAATAATAAAAAATTTTTCTATAAATTATGACCAAATTTCATTATTTCCATGATCAGT 471
AMC_2 TTAAACTTAATAGTTACAATTATAATAATAAAAAATTTTTCTATAAATTATGACCAAATTTCATTATTTCCATGATCAGT 480
AMM_1 TTAAATTTAATAGTTACAATTATAATAATAAAAAATTTTTCTATAAATTATGACCAAATTTCATTATTTCCATGATCAGT 477
AMM_2 TTAAATTTAATAGTTACAATTATAATAATAAAAAATTTTTCTATAAATTATGACCAAATTTCATTATTTCCATGATCAGT 475
AMM_3 TTAAATTTAATAGTTACAATTATAATAATAAAAAATTTTTCTATAAATTATGACCAAATTTCATTATTTCCATGATCAGT 473

Majority TTTTATTACAGCAATTTTATTAATTATATCATTACCTGTATTAGCTGGAGCAATTACTATATTATTATTTGATCGAAATT

---------+---------+---------+---------+---------+---------+---------+---------+

490 500 510 520 530 540 550 560

---------+---------+---------+---------+---------+---------+---------+---------+

AMC_1 TTTTATTACAGCAATTTTATTAATTATATCATTACCTGTATTAGCTGGAGCAATTACTATACTATTATTTGATCGAAATT 551

```
AMC_2     TTTTATTACAGCAATTTTATTAATTATATCATTACCTGTATTAGCTGGAGCAATTACTATACTATTATTTGATCGAAATT     560
AMM_1     TTTTATTACAGCAATTTTATTAATTATATCATTACCTGTATTAGCTGGAGCAATTACTATATTATTATTTGATCGAAATT     557
AMM_2     TTTTATTACAGCAATTTTATTAATTATATCATTACCTGTATTAGCTGGAGCAATTACTATATTATTATTTGATCGAAATT     555
AMM_3     TTTTATTACAGCAATTTTATTAATTATATCATTACCTGTATTAGCTGGAGCAATTACTATATTATTATTTGATCGAAATT     553

Majority  TTAATACATCATTTTTTGATCCTATAGGAGGTGGAGATCCAATTTTATATCAACATTTATTTTGATTTTT----------
          ---------+---------+---------+---------+---------+---------+---------+---------+
                  570       580       590       600       610       620       630       640
          ---------+---------+---------+---------+---------+---------+---------+---------+
AMC_1     TTAATACATCATTCTA----GCTTTATGATCTGGA-----------ATACTAGGAT----------C------------     593
AMC_2     TTAATACATCATTTTTCGATCCTATAGGAGGTGGAGATCCAATTCTTTATCAACATTTATTTTGATTTTT----------     630
AMM_1     TTAATACATCATTTTTTGATCCTATAGGAGGTGGAGATCCAATTTTATATCAACATTTATTTTGATTTTT----------     627
AMM_2     TTAATACATCATTTTTTGATCCTATAGGAGGTGGAGATCCAATTTTATATCAACATTTATTTTGATTTTTTTATATTATT     635
AMM_3     TTAATACATCATTTTTTGATCCTATAGGAGGTGGAGATCCAATTTTATATCAACATTTATTTTGATTTTT----------     623

Majority  ---------------------------CATCAATGAGACTTATTATTCGAATAGAATTAAGATCC-CCAGGATCATGAA
          ---------+---------+---------+---------+---------+---------+---------+---------+
                  650       660       670       680       690       700       710       720
          ---------+---------+---------+---------+---------+---------+---------+---------+
AMC_1     ----------------------------ATCAATGAGACTTATTATTCGAATAGAATTAAGATCC-CCAGGATCATGAA     643
AMC_2     ---------------------------CATCAATGAGACTTATTATTCGAATAGAATTAAGATCC-CCAGGATCATGAA     681
AMM_1     ---------------------------TATCAATGAGACTTATTATTCGAATAGAATTAAGATCC-CCAGGATCATGAA     678
AMM_2     CTAGCTTTATGATCTGGAATATTAGGTTCATCAATGAGACTTATTATTCGAATAGAATTAAGATCT-CCAGGATCATGAA     714
AMM_3     -----------------------------TGGAGCTAGTTTATTATTATATCATTAACGAGGTCGATTATGTCCATTAC     673

Majority  TTAACAATGATCAAATTTATAATACAATTGTTACTAGTCATGCATTCCTAATAATTTTTTTTATAGTTATACCATTTTTA
          ---------+---------+---------+---------+---------+---------+---------+---------+
                  730       740       750       760       770       780       790       800
          ---------+---------+---------+---------+---------+---------+---------+---------+
AMC_1     TTAGCAATGATCAAATTTATAATACAATTGTTACTAGTCATGCATTCCTAATAATTTTTTTTATAGTTATACCATTTTTA     723
AMC_2     TTAACAATGATCAAATTTATAATACAATTGTTACTAGTCATGCATTCCTAATAATTTTTTTTATAGTTATACCATTTTTA     761
AMM_1     TTAGCAATGATCAAATTTATAATACAATTGTTACTAGTCATGCATTCCTAATAATTTTTTTTATAGTTATACCATTTTTA     758
AMM_2     TTAATAACGATCAAATTTATAATACAATTGTTACTAGCCACGCATTTCTAATAATCTTTTTTATAGTTATACCATTTCTA     794
AMM_3     T--ATATTAATTA--TTT-TAACGACATTATTTTTGACTA-GTACCTTTATTACTTTAAACCAGTATTAAAT-ATCTTT-     745
```

```
Majority ATTGGAGGATTTGGAAATTGGC-TTATTCCTTTAA--TACTAGGAT--CACCTGATATAGCATTCCCCCGAATAAATAAT
         ---------+---------+---------+---------+---------+---------+---------+---------+
                810       820       830       840       850       860       870       880
         ---------+---------+---------+---------+---------+---------+---------+---------+
AMC_1    ATTGGAGGATTTGGAAATTGGC-TTATTCCTTTAA--TACTAGGAT--CACCTGATATAGCATTCCCCCGAATAAATAAT    798
AMC_2    ATTGGAGGATTTGGAAATTGGC-TTATTCCTTTAA--TACTAGGAT--CACCTGATATAGCATTCCCCCGAATAAATAAT    836
AMM_1    ATTGGAGGATTTGGAAATTGGC-TTATTCCTTTAA--TACTAGGAT--CACCTGATATAGCATTCCCCCGAATAAATAAT    833
AMM_2    ATTGGAGGATTTGGAAATTGGC-TTATTCCTTTAA--TACTAGGAT--CACCTGATATAGCATTTCCCCGAATAAATAAT    869
AMM_3    -TTAAAAAATAATAATATTAACATTGATAATTTAAATTACTAGGATATTAACTCCTTTAAGGACTATATACTTCTCTTTT    824

Majority ATTAGATTTT-GATTACTTCC---TCCCTCATTATTTATACTTTTATTAAGAAATTTATTTTATCCAAGACCAGGAACTG
         ---------+---------+---------+---------+---------+---------+---------+---------+
                890       900       910       920       930       940       950       960
         ---------+---------+---------+---------+---------+---------+---------+---------+
AMC_1    ATTAGATTTT-GATTACTTCC---TCCCTCATTATTTATACTTTTATTAAGAAATTTATTTTATCCAAGACCAGGAACTG    874
AMC_2    ATTAGATTTT-GATTACTTCC---TCCCTCATTATTTATACTTTTATTAAGAAATTTATTTTATCCAAGACCAGGAACTG    912
AMM_1    ATTAGATTTT-GATTACTTCC---TCCCTCATTATTTATACTTTTATTAAGAAATTTATTTTATCCAAGACCAGGAACTG    909
AMM_2    GTTAGATTTT-GATTACTTCC---TCCCTCATTATTAATACTTTTATTAAGAAATTTATTTTACCCAAGACCAGGAACTG    945
AMM_3    TTAACGTTTTAGATGACTTCCACTTCTTACTATATTTATACGACTATTACCACCTATATGACAAGTAGGTCAAGGACCAG    904

Majority GATGAACAGTATATCCACCATTATCAGCATATTTATATCATTCTTCACCTTCAGT---AGATTTTG-CAATTTTTTCTCT
         ---------+---------+---------+---------+---------+---------+---------+---------+
                970       980       990      1000      1010      1020      1030      1040
         ---------+---------+---------+---------+---------+---------+---------+---------+
AMC_1    GATGAACAGTATATCCACCATTATCAGCATATTTATATCATTCTTCACCTTCAGT---AGATTTTG-CAATTTTTTCTCT    950
AMC_2    GATGAACAGTATATCCACCATTATCAGCATATTTATATCATTCTTCACCTTCAGT---AGATTTTG-CAATTTTTTCTCT    988
AMM_1    GATGAACAGTATATCCACCATTATCAGCATATTTATATCATTCTTCACCTTCAGT---AGATTTTG-CAATTTTTTCTCT    985
AMM_2    GATGAACAGTATATCCACCATTATCAGCATATTTATATCATTCTTCACCTTCAGT---AGATTTTG-CAATTTTTTCTCT   1021
AMM_3    AACCCATTTTATTTAAAGAATTATTTTCATAATTATTACTCCCTCCTTCATTAGTTTTAGATTGTAATAAATAAGCCCCT    984

Majority TCAT-ATAT----CAGGAATTTCCTCAATTATAGGATCA-TTAAA--CTTAATAGTT--ACAATTATAATAATAAAAAAT
         ---------+---------+---------+---------+---------+---------+---------+---------+
               1050      1060      1070      1080      1090      1100      1110      1120
```

```
         ---------+---------+---------+---------+---------+---------+---------+---------+
AMC_1    TCAT-ATAT----CAGGAATTTCCTCAATTATAGGATCA-TTAAA--CTTAATAGTT--ACAATTATAATAATAAAAAAT    1020
AMC_2    TCAT-ATAT----CAGGAATTTCCTCAATTATAGGATCA-TTAAA--CTTAATAGTT--ACAATTATAATAATAAAAAAT    1058
AMM_1    TCAT-ATAT----CAGGAATTTCCTCAATTATAGGATCA-TTAAA--CTTAATAGTT--ACAATTATAATAATAAAAAAT    1055
AMM_2    TCAT-ATAT----CAGGAATTTCCTCAATTATAGGATCA-TTAAA--TTTAATAGTT--ACAATTATAATAATAAAAAAT    1091
AMM_3    TTACGATATAGTCCACTAGGATCATAATTTCCTTATTCGGTTAAAGGTTTAGGAGGTTAATCTTTACCATATTGATATTT    1064

Majority TTTTCTATAAATTATGACCAAATTTCATTATTTCCATGATCAGTTTTTATTACAG-CAATTTTATTAATTA-TATCATTA
         ---------+---------+---------+---------+---------+---------+---------+---------+
               1130      1140      1150      1160      1170      1180      1190      1200
         ---------+---------+---------+---------+---------+---------+---------+---------+
AMC_1    TTTTCTATAAATTATGACCAAATTTCATTATTTCCATGATCAGTTTTTATTACAG-CAATTTTATTAATTA-TATCATTA    1098
AMC_2    TTTTCTATAAATTATGACCAAATTTCATTATTTCCATGATCAGTTTTTATTACAGGCAATTTTATTAATTA-TATCATTA    1137
AMM_1    TTTTCTATAAATTATGACCAAATTTCATTATTTCCATGATCAGTTTTTATTACAG-CAATTTTATTAATTA-TATCATTA    1133
AMM_2    TTTTCTATAAATTATGACCAAATTTCATTATTTCCATGATCAGTTTTTATTACAG-CAATTTTATTAATTA-TATCATTA    1169
AMM_3    TTTCTAATAATCTTTACGCACCGATCATTGTTAACATAA----TATTTAAACTAG-CAAT--AATTAAGTACTAGGACCT    1137

Majority CCTGTATTAGCTGGAGCAATTACTATACTATTATTTGATCGAAATTTTAATACATCATTTTTCGATCXTATXXXAXXXXX
         ---------+---------+---------+---------+---------+---------+---------+---------+
               1210      1220      1230      1240      1250      1260      1270      1280
         ---------+---------+---------+---------+---------+---------+---------+---------+
AMC_1    CCTGTATTAGCTGGAGCAATTACTATACTATTA    1131
AMC_2    CCTGTATTAGCTGGAGCAATTACTATACTATTATTTGATCGAAATTTTAATACATCATTTTTCG    1201
AMM_1    CCTGTATTAGCTGGAGCAATTACTATACTATTATTTGATCGAAATTTTAATACATCATTGTTCGATCCTATAGGAGGTGG    1213
AMM_2    CCTGTATTAGCTGGAGCAATTACTATATTATTATTTGATCGAAATTTTAATACATCATTTTTTGATCCTATAGGAG    1245
AMM_3    CTAGAATTAAGATAAGC--TTATTATTCAGAGTAACTACTTGGATTATAAGGTCTAGTATTTCGATCTTATTATATGTTC    1215
```
